# The History and Challenges of Women in Genetics: A Focus on Non-Western Women

**DOI:** 10.3389/fgene.2021.759662

**Published:** 2021-11-25

**Authors:** Hadeel Elbardisy, Malak Abedalthagafi

**Affiliations:** Genomics Research Department, Saudi Human Genome Project, King Fahad Medical City, King Abdulaziz City for Science and Technology, Riyadh, Saudi Arabia

**Keywords:** women in science, non-western, genetics, gender, career

## Abstract

“Women in much of the world lack support for fundamental functions of a human life.” This truthful portrait was pointed out by Martha Nussbaum in her book “*Introduction: Feminism & International Development.”* Throughout history, gender inequality has been persistent in many aspects of life, including health and empowerment. Unfortunately, this inequality has not been excluded from the field of science. Perpetual assumption that women’s absence or restriction to secondary roles in various disciplines is an acceptable law of nature misrepresents women’s contribution to science and maintains hurdles for participation in the future. According to a recent UNESCO’s report, women make up only 30% of researchers worldwide. But despite all the obstacles, women made major contributions with discoveries that shaped the progress in many scientific fields. In the field of genetics, Rosalind Franklin is an example of unwittingly compromised women’s scientific achievements. Franklin was an expert in X-ray crystallography; her data, especially the “photo 51,” was critical to James Watson and Francis Crick along with their own data to publish the discovery of the double helix DNA structure in 1953. Her contribution was acknowledged posthumously in Watson’s memoir in 1968. Barbara McClintock was a 20th century American cytogeneticist who remains up to date the only woman receiving an unshared Nobel prize in Physiology or Medicine. McClintock dedicated her work to cytogenetics and discovered the phenomenon of mobile genes. Her research was initially subjected to skepticism in the 1950s. It was not until the late 1960s that the community realized the significance of McClintock’s discovery. The history of science is occupied with a myriad of similar tales of such inspiring women that, after tremendous struggles, thrived and achieved breakthroughs in their respective fields. It is prominent our limited knowledge of women’s experience and struggle in science in non-western world. Addressing the stories of this outstanding minority is critical to expand the understanding of the gender disparity factors embedded in diverse cultures. In this article, we attempt to put the spotlight on some fascinating non-western women and their significant contributions to the field of genetics.

## Women in Genetics

Today women are able to succeed in a still male-dominated science community and prove their pivotal roles, although there are still many significant obstacles present, including social norms, political systems, and religious backgrounds resulting in gender disparities. Policymakers’ lack of awareness reform these obstacles sterner with less priority given to address the gender gap (Andres, 2011). Women under representation in the science field could also be rooted to a diversified restrain faced in higher education, career path, working environment, role stereotypes and the family work balance (Handelsmann et al., 2005). In a recent study, gender inequality in scientific careers was analyzed through a large scale longitudinal bibliometric analysis. The analysis showed that women starting their publication career have proportionally increased over the course of recent years from 30% (2000) up to almost 40%. Yet, male researchers often publish an average of 15–20% more than female researchers (Boekhout et al., 2021). Also, the tendency that women discontinue publications was slightly higher than men–an indication of their dropout and it revealed that about 25% of men are more often the last authors than women of similar career years–an indication of more senior positions (Sanderson, 2021; Boekhout et al., 2021). This gender imbalance is also presented in high prestigious research awards as Nobel prize, etc. Between 2001 and 2020, a total of 2011 men were awarded compared to 262 women only (Watson, 2021). Worth mentioning, female scientists annual share of awards has raised from 6% up to 19% between 2016 and 2020 (Meho, 2021). But the gender disparity is still consistent if we consider the average numbers of full-time female researchers particularly in biological, life sciences, computer science and mathematics (Meho, 2021). In 2020, Emmanuelle Charpentier and Jennifer Doudna were announced winners of Nobel prize in chemistry for the development of the CRISPR/Cas9 genome editing methodology, a revolutionary novel tool for gene editing (Nobel Prize, 2020). The CRISPR/Cas9 system is present naturally in archaea and bacteria, acting as a defensive shield against pathogens and thus providing immunity against these viruses and plasmids (Terns and Terns, 2011). The system works as precise genetic scissors, allowing double strand cleavage at specific regions in the DNA that are complementary to a subset of mature CRISPR RNAs (crRNAs). The crRNAs base pairs form a dual RNA with trans-activating crRNAs (TracrRNA). These dual RNAs are able to target specific sites and sequences within the genomic DNA permitting precise genome editing (Jinek et al., 2012). This CRISPR technology could be applied for genome-wide screening, editing of gene coding sequences, epigenome editing and transcriptional regulation. Thus, the CRISPR technology represents a promising therapeutics tool for genetic disorders and targeted cancer therapies (Barrangou and Doudna, 2016). Moreover, CRISPR genome editing represents an exciting new tool for wide range of applications in the agricultural, food, and industrial biotechnology sciences (Barrangou and Doudna, 2016). Throughout the awards history, this is the first time for two women to share a Nobel prize in chemistry (Rincon, 2020). In an interview with C and EN, Doudna said “It certainly makes me happy that it could be the case that because two women were involved in the early days of CRISPR that we could have established a culture that is welcoming to other women in the field. That’s kind of cool.” This was her answer to if she believes that CRISPER could be the unique tool that welcomes and pave the road for female scientists (Satyanarayana, 2020).

By the end of 2019, the world witnessed the emergence of a new coronavirus leading to the ongoing COVID-19 pandemic. Unexpectedly people’s daily life came to a pause with lockdowns imposed all over the world (Chams et al., 2020). History portrays human survival against several outbreaks and pandemics affecting thousands to millions lives. Although our advancements in technology and medicine are of critical importance, we came to realize our vulnerability towards this novel pathogen. The escalating rates in mortality and morbidity alerted the science community to act rapidly to develop strategies against this pandemic (Hu et al., 2021). From understanding the virus, it’s pathogenesis, methods of diagnosis and description of clinical manifestations to genome sequencing and using molecular and genetic data to seek a therapeutic route or effective vaccination, scientists were in a race against the pandemic spread of COVID-19. Several women were at the frontline of developing COVID-19 vaccines (Bora, 2021). Professor Sarah Gilbert, the women behind the Oxford/AstraZeneca vaccine, led a whole team in a race to develop a vaccine and push for the preclinical and clinical testing (BBC, 2020; Lane, 2020). Her previous work on malaria vaccine research, which focused on developing vaccines that preferably trigger a T-cell response over triggering B-cell antibody responses alone paved the route for creating vaccines that contain specific antigens within the viral host, a technology known as recombinant viral vector vaccines which outweighed the risk concerns associated with traditional live attenuated vaccines. Gilbert’s team focused on this technology in their work on the COVID-19 vaccine (Lane, 2020). Over a year, the Oxford/AstraZeneca vaccine was approved and now it is being administrated worldwide to impede the virus spread (WHO, 2020). A work of excellence credited to the oxford team which composed of two third female researchers led by Dr Gilbert (PA Media, 2021). At a point of her life, Dr Gilbert did consider leaving science for good especially during her doctorate study. Back then, she was fueled with energy to gain experience from diverse disciplines but was faced with “one subject focus” ideology. Luckily for us, she did not leave science, although her career got more challenging after giving birth to triplets (BBC, 2020). She is a true example of a committed scientist and a devoted mother who excelled at both. Diverse heroines also had outstanding roles in the development of novel vaccination strategies to combat COVID-19 (Bora, 2021; Romero et al., 2020). Dr Kizzmkia Corbertt, an African American scientist, had an eloquent role in the development of Moderna’s COVID-19 vaccine. She also took a deepened commitment to outreach communities of color to alleviate the vaccine skepticism (Subbaraman, 2021). Dr Özlem Türeci, a notorious scientist, physician and entrepreneur who was instrumental in developing the first FDA approved mRNA vaccine (BioNTech-Pfizer) within just a year (Bryer, 2021). Dr Türeci, the descent of Turkish immigrants and the cofounder of BioNTech company, confronts the gender inequality by adopting a balanced workforce in her company with 54% being females. She credited the rapid vaccine release to this equitable workforce (PA Media, 2021). These scientists and many more show the tangible impact of women’s inextricably role during the global crisis. In this context, the power of role models for young girls is critical. Mattel company released six Barbie dolls to honor women in science and their contributions in the fight against COVID-19 in last August (Joly and Shea, 2021)- a splendid step to reform the image of an unrealistic plastic doll into a doll depicting real and successful women scientists as role model for our future generations of female scientists.

## Non-Western Women in Genetics

A study discussing the gender inequality paradox in STEM fields pointed out that women living in non-western countries with a greater gender equality gap are more likely to be engaged in STEM as a result of the society pressure and pursuing an improved overall quality of life (Stoet and Geary, 2018). The UNESCO Institute for Statistics fact sheet released in June 2020 showed that women in R&D represent 25% for East Asia and the pacific, 23.1% for South and West Asia (∼50%) and 40.9% for Arab States, while only 32.9% of researchers were female in North America and Western Europe (Table 1) (UNESCO Institue for Statistics, 2020). Here, we present selected recounts of nonwestern women from these regions, our selection is based on several reasons. To begin with, regions like East Asia and the Pacific progress to narrow gender disparities in diverse fields is tardy over the years with 2.5% improvement from 2006 to 2019, a concerning alarm for a region of one of highest women percentages globally (1.13 billion women) (World Economic Forum, 2020). On contrary, Southeast Asia region attains a grappling progress towards narrowing the gender gap (Bekhouche, 2013). Important factors mastered this improvement. For example, Singapore ranks number one as one of the safest location for women in Asia Pacific (Evlanova, 2019). With a powerful law protecting women’s rights and a labor force comprised of 60% women, resources to fair treatment for women outstand clear in Singapore (Setianto, 2020). To bring visibility to today’s women’s position in these regions, we selected three stories particularly in the field of genetics representing South Korea, Singapore, and Thailand.

**TABLE 1:** The average percentage of females involved in R&D positions (full time and Part time jobs) in different regions globally, according UIS fact sheet (UNESCO Institue for Statistics 2020).

Region	Average percentage (%)
Central Asia	48.5
Latin America and the Caribbean	45.8
Arab States	40.9
Central and Eastern Europe	39.0
North America and Western Europe	32.9
Sub-Saharan Africa	31.1
East Asia and the Pacific	25.0
South and West Asia	23.1

Narry Kim, a South Korean molecular cell biologist, has made critical contributions to our understanding of RNA biology (The Royal society, 2021). Her journey began with receiving her BSc from Seoul National University in 1992 and her PhD from Oxford University in 1998, where her research focused on retroviral proteins and their role in constructing gene transfer vectors. Her post-doctoral research took place in the laboratory of Gideon Dreyfuss at the University of Pennsylvania in Philadelphia where she worked on studying mRNA surveillance pathways (Cell Symposia, 2019). Organisms have these pathways to ensure the fidelity of mRNA during its biogenesis (Hoof and Wagner, 2011). In 2001, She returned to Seoul National University (SNU) as a faculty member, and by 2013 she became a SNU distinguished Professor (Seoul National University, 2021). Her research team is focused on the control of gene regulation by RNA. RNA molecules have a crucial role in post-translational gene regulation and are key players in some diseases (Mattick, 2011). Understanding their pathways and mechanisms of action promises novel insights and the development of improved therapeutic solutions, for example in cancer therapies and stem cell engineering (Narry Kin Lab, 2021). Three main research directions [microRNA (miRNA), RNA tails, and RNA binding proteins (RBP)] are her laboratory’s main topics of interest (Seoul National University, 2021). Dr. Kim’s group was able to elucidate the mechanisms of miRNA biogenesis through two sequential steps: pre-miRNAs generation in the nucleus and processing of these pre-miRNA into mature miRNAs in the cytoplasm (Lee et al., 2002). They identified several key factors within the pathway such as DROSHA, DGCR8, RNA polymerase II and a terminal nucloetidyl transferases known as uridyltransferases (The Royal society, 2021). Her research group was able to identify other key factors, including the RNA binding proteins Lin28a and Lin28b important in stem cell programming (Heo et al., 2008). They also developed a novel experimental tool called TAIL-seq for genome-wide screening for mRNAs tails [poly(A) and 3′ end modification]. This tool has helped to reveal the roles of these mRNA tails in diseases (Chang et al., 2014). In an approach to understand the complexity of RNA binding proteins (RBP), Kim’s lab has founded a proteomic facility to develop novel techniques to scrutinize RBP networks (Seoul National University, 2021). Narry Kim was recognized nationally and internationally by the scientific community for her significant contributions. In 2007, she was named Woman Scientist of the Year by the Ministry of Science and Technology of South Korea. She received the L’Oreal-UNESCO Women in Science Award in 2008. By that time, Dr Kim was one of the very few female scientists being recognized in the Asian pacific region. In an interview upon her winning, Dr Kim stated the limited independent positions and almost no leadership positions for female researchers must not endure. She already witnessed the loss of talented females in the field and called for more efforts to be done by the government to push women in science ( Kim, 2008 ). She was named a National Honor Scientist by the Ministry of Education, Science and Technology in 2010. In 2013, Dr. Kim was awarded the S-Oil Leading Scientist of the Year Award, the Korea S&T Award, and the Gwanak Grand Prize Honor Sector by Seoul National University. In 2017, she received the Chen Award. In 2019, she received the Asian Award in Medicine. She was elected as a Foreign Associate of the prestigious European Molecular Biology Organization (EMBO) in 2013, a Foreign Associate of the US National Academy of Science (NAS) in 2014, and Member of Korean Academy of Science and Technology (KAST) in 2014 (Narry Kin Lab, 2021). Currently, she is the director of the RNA research at the Institute for Basic Science (IBS) leading the research on high resolution mapping of the new coronavirus RNA that will help in finding more accurate strategies against COVID-19 (Kim 2020).

Chanchao Lorthongpanich is a young leading principal investigator and developmental stem cell biologist at the Siriraj Center of Excellence for Stem Cell Research in Bangkok, Thailand (SiSCR, 2021). Early on she understood the urgency of resolving the problem of limited blood supply in hospitals which can be life-threatening for patients. Dr. Lorthongpanich and her research team developed an alternative intervention for blood supply shortage in hospital patients by establishing an *in vitro* production system (UTAR, 2021). They were able to revert differentiated adult cells from patients to a stem cell state and therefore generate patient-specific induced pluripotent stem cells (iPSCs). The adult cells were retrieved from patients suffering from paroxysmal nocturnal hemoglobinuria (PNH), a disease developing from genetic mutations specifically in hematopoietic stem cells, resulting in severe hemolytic anemia, thrombosis and peripheral blood cytopenia (Brodsky, 2014). By utilizing iPSCs to generate autologous hematopoietic stem cells (HSCs), they created HSC without the disease-causing mutation which thus can then be used as a conventional treatment available for PNH. This method of retrieving cells from a patient and directing them to differentiate into HSCs avoids the complications resulting from allogenic HSCs transfusions, lack of matching HLA donors and post-transplant complications (Phondeechareon et al., 2016). In an interview with the journal *Nature*, she referred to her current focus on developing human platelets inside the laboratory, a lifesaving approach to overcome the continuous shortage of platelet donors, especially in Thailand (Nogrady, 2019). Enhancing the large scale production of platelets from hematopoietic stems cells holds the potential of providing abundant donor-independent platelets that would be lifesaving for many patients with critical conditions (Bangkok UNESCO, 2018). She advocates for Thailand to be an ideal destination to establish biotechnology factories due to the decreased expenses of living (Nogrady, 2019). Lorthongpanich addresses the burden of inadequate funding during her career but a deepened commitment to science and her country steered her will. For that, Chanchao Lorthongpanich was awarded the L’Oreal-UNESCO for Women in Science Awards in 2018 for her outstanding contributions to stem cell research.

Yue Wan is a senior research scientist at the Genome Institute of Singapore, a junior principal investigator at the Agency for Science, Technology and Research (A*STAR), and an adjunct assistant professor in the Department of Biochemistry at the National University of Singapore (Wan, 2021). Her research interest is focused on understanding RNA functional structures and their roles in the regulation of cellular processes. Her interests include the impact of RNA structures on cellular states, RNA interactions networks in different organisms, and the genome of RNA viruses to better understand infectious diseases and their impact on human health (Wan, 2021). Dr. Wan has developed several ingenious novel laboratory tools for the study of RNA. She and her team were able to reveal the intramolecular and intermolecular RNA-RNA interactions in eukaryotes utilizing a high-throughput approach called Sequencing of Psoralen cross-linked, Ligated, And Selected Hybrids (SPLASH) (Aw et al., 2016). They showed that SPLASH could be an informative tool in understanding the complexity of eukaryotes transcriptomes, providing a deeper understanding of how RNAs interact with each other and the surrounding cellular molecules (Aw et al., 2016). Wan and her team also developed a new technology called Parallel Analysis of RNA Structures (PARS) to determine thousands of RNA structure simultaneously, a method that enables better insight of RNA structures to fully comprehend their function in cellular states (Wan et al., 2011; Kertesz and Yue, 2010). Recently, they published their work on developing a nanopore sequencing method called PORE-cupine to determine distinct RNA isoforms of the same gene and their ability to adapt different structural conformations (Aw et al., 2021). Wan was recognized for her research in Singapore and abroad. In 2015, she received the Genome Web Young Investigator Award from the Singapore National Academy of Science, and the Young Scientist Award and EmTech MIT TR35 Asia Honoree, an award given to promising innovators under the age of 35 organized by MIT Technology Reviews (The Branco Weiss Fellowship, 2021). She was the first Singaporean scientist to win the Branco Weiss Fellowship granted by a Swiss based philanthropic organization which every year awards 10 outstanding scientists (Asian Scitentist Newsroom, 2014). In 2016, she was awarded the Ten Outstanding Young Person Award Finalist in Singapore, an A*STAR Investigator position, and the 2016 L’Oréal Singapore For Women in Science National Fellowship (Asian Scientist Newsroom, 2016). As a young mother of two, she strongly supports a family friendly policy to be offered to female researchers that would spin their productivity and wellness (A*Star Talent Times, 2021).

Accounts of an older generation of women scientists shows how influential women have been in the field of science. With a false myth of botany being imposed as an “amusement” for women with restricted role as home gardeners, plant gatherers and housewives’ herbalist. Studying the science of plants was viewed as a field being exclusively owned by men (Howard, 2001). A lackluster belief that encouraged us to choose and acknowledge Dr. Archana Sharma, the godmother of Botany. Dr. Sharma, being born to an academic family in India, completed her B.Sc. from Bikaner University and pursued her master’s and Ph.D. degrees in the Department of Botany at the University of Calcutta (Sopory, 2009). In 1960, she became the second women receiving her doctorate in botany (D.Sc.) from the University of Calcutta, one of the oldest universities in India (Pathiki et al., 2015). Her research was not only focused on botany, but it also expanded to cytogenetics, human genetics, and environmental mutagenesis. After her education, Dr. Sharma became a faculty member in the department of Botany at the University of Calcutta. By 1972, along with her husband professor AK Sharma, she was appointed a professor of genetics in the Center of Advanced Studies in Cell and Chromosome Research after the foundation of a school of cytogenetics (Shah, 2018). In 1981, she was promoted to Head of the Department of Botany at the University of Calcutta. Dr. Sharma inventing revolutionary novel methods for visualizing chromosome structures that soon became gold standard techniques for plant cell and chromosome research worldwide (Sharma and Archana, 1956). Later, Sharma and her husband published a book summarizing their research and findings on chromosomes, aptly entitled: “Chromosome techniques–theory and practice” (Sharma and Sharma, 1980). This represented an informative repository of molecular and histological techniques, including pre-treatment and hypotonic techniques, fixation, staining and processing of cells, understanding chromosome structures and analysis after culture of cells, and various techniques to visualize the banding patterns of chromosomes (Sharma and Sharma, 1980). This textbook remains very popular as one of the standard curriculums in the field of chromosome research and botany. Prof. Sharma’s laboratory produced multiple novel discoveries: her findings of the speciation in the vegetative reproduction of plants raised a new concept of how new species evolve after plants propagate asexually through regular inconsistent chromosome mosaicism (Pathiki et al., 2015). Furthermore, she studied methods of inducing cell divisions in mature nuclei and the causes of polyteny in plants. In human genetics, she focused on comparative studies in genetic polymorphism between normal populations versus those with pathological conditions and investigated the sex abnormalities that are prominent among the Indian population (Nigli et al., 1980). Elevated levels of environmental pollution due to industrial discharges and increased use of pesticides alerted scientists to investigate the effects of this pollution on the Indian population. Her group investigated the impact of pesticides and heavy metals on various biological systems, and the clastogenic impact and hazardous effects on chromosomal abnormalities, mitosis inhibition and cell division (Giri et al., 1984; Agarwal et al., 1990; Mukherjee and Sharma, 1987). She established *The Nucleus*, an international journal for cytology and allied topics of cell and chromosome research (Sopory, 2009). Her research and contributions were recognized nationally and internationally. She was a fellow of the Indian National Science Academy in 1977 and was elected President of the Indian Botanical Society in 1989. Between 1986 and 1987 she was the general president of the Indian Science Congress Association. In 1990, she was a member of the International Academy of Science in Germany (Pathiki et al., 2015). She was awarded the Shanti Swarup Bhatnagar Prize for Science and Technology in 1976. She was the recipient of the Padma Bhushan Award in 1984 and received the Women Scientist Award and the Ashutosh Mukherji Medal by the Indian Science Congress Association in 1999 (Pathiki et al., 2015). Moreover, she was a policy maker in the Indian government as she was a member of the Science and Engineering Research Council and the Environmental Research Council. In addition, she was also a member of the panel for the cooperation with the UNESCO (Sopory, 2009).

The position of African women in STEM remains a prominent concern as poverty, health and education inequality are more potent barriers in Africa. An embedded culture of women’s role as an exclusive family caregiver plays a pivotal role of young girls drop out after primary education (Andres, 2011). The racial discrimination reinforces the issue especially when it comes to fair opportunities in funded scholarships, health equity and workforce infrastructure (Ighodaro et al., 2021). In 2015, the African Union announced the Year of Women’s Empowerment and Development Towards Africa Agenda 2063. This Agenda had the goal to further develop the Science, Technology, and Innovation Strategy for Africa 2024 into an initiative to advocate for women’s inclusion in these areas (Muthumbi and Sommerfeld, 2015) and to promote women in Africa to participate and become key members in the field of science. We selected our upcoming scientist from Nigeria, as it is one of the highest population country in the region with accentuated under-representation of women in research and a paucity of them in senior positions as well (WHO, 2020). Alongside with poverty, religious and cultural obstacles mentioned above (Andres, 2011). We share the inspiring story of Adeyinka Falusi, a scientist that devoted her career to human genetics, bioethics and inherited hematological diseases in her home country. Her early inspiration came from a childhood friend, Grace Olaniyan, and her interest in science. Out of curiosity, she read Grace’s science textbooks and became deeply interested in the topics discussed. Adeyinka Falusi earned her M.Phil. and PhD at the University of Ibadan in 1986, with her research dedicated to elucidating the various types of anemia and sickle cell diseases (SCD) as inherited genetic disorders in the Nigerian population. Along with her team, she was able to screen for and discover exclusive genetic markers for sickle cell anemia (kulozik et al., 1986), a remarkable paradigmatic shift in the prevalence of published sickle cell variants. Her research extended to the molecular epidemiology of the compounding impact of several hematological diseases including Malaria and Thalassemia (Higgs et al., 1986; Fey et al., 1990). She also studied the genetics of breast cancer among Nigerian women (Yonglan et al., 2018), which was one of the first and foremost genetic studies conducted in the Nigerian population. Her work in the field of SCD granted her a L’OREAL UNESCO Outstanding Woman of Science Award in 2001, and she received the Rare Gem Award in the Category of Science and Technology in 2003. She was awarded the National Productivity Order of Organization for Women in Science for Developing Worlds (Adeyinka, 2021) and received a Vocational Excellence Award for Impact in Science in 2014. Recognizing the lack of community awareness for SCD, she founded the Sickle Cell Association of Nigeria (SCAN) and later the Sickle Cell Hope Alive Foundation (Adeyinka, 2021; The Network of African Science Academies, 2017). In an interview, she recounted that 1 day her director encouraged her to engage with the community and share her knowledge with the people of Nigeria. That day she went to the local Yemetu and Adeoyo hospitals and started spreading awareness, support, and information, which later morphed into the establishment of SCHAF where she donated her retirement money (Gesinde, 2020). Under her leadership, the foundation promoted community awareness with free parent’s handbooks for sickle cell patients (The Network of African Science Academies, 2017). One of her other notable contributions is her eminent role in research bioethics. She extensively worked on the development of institutional ethics review board at her university that grew to an ethical model review board nationally. From 2001 to 2005 she served as the chairperson of the University of Ibadan/University Collage Hospital Institutional Review Committee, where she was keen to restructure the review committee and establish the first guidelines for ethical reviews in the University. She was the country coordinator for Nigeria in the Networking for Ethics of Biomedical Research in Africa (NEBRA), promoting research ethics in central and western Africa from 2005 to 2006. Her work in bioethics was awarded with the EDCTP award for her outstanding role in establishing research ethics review boards in Africa. Dr. Falusi also established the Nigerian Bioethics Initiative (NIBIN) (African Success, 2009), and serves as a truly inspiring example of an African women scientist. She was privileged with support from her husband and family, an advantage not common in Nigeria. Although, she sacrificed many years prior joining academics to take care of her five children yet remained persist to achieve her dream and utilize her knowledge to help Nigerian people ( Gesinde, 2020 ).

Although women in the Middle East have made progress in STEM fields and R&D, with the percentage of women exceeding 40% (UNESCO Institue for Statistics, 2020), there are still challenges faced by women in this cultural environment. Social norms, gender inequality, religious background, early marriage, and childbirth are glimpses of the many obstacles’ women are confronted with in the Middle East. These societal obstacles, together-a lack of equal opportunities, salaries and support, cause woman to still struggle to balance a healthy work–family life. The dominating ideology that men should be the primary source of income is one of the root causes of this inequality and salary gaps. It is estimated that three out of four women do not work even after completing their education (Gatti et al., 2013). In some countries like Egypt, Jordan and Tunisia women with higher education degrees suffer lower employment opportunities than women with lower educational levels. On the contrary, Gulf countries are promoting employment opportunities for educated women (International labour office, 2016). A statement that influenced our selection from this region to represent fruitful stories from both proportions. On the bright side, there is a rising awareness for the need to support employment of women, with governments pushing women’s enrollment in education, labor force and managerial positions (Patel, 2019).

We here present some Arab women scientists that made remarkable contributions in the field of genetics in the Middle East. Nadia Sakati is a distinguished pediatrician who revolutionized genetics in Saudi Arabia. She had a dream of being a doctor wearing a white gown since she was in grade 8 (Takreem, 2018). By 1965, she had completed her medical degree from the Medical School of Damascus University. Dr. Sakati then worked as a pediatric resident at the American University of Beirut and the Jackson Memorial Hospital in Miami in 1966. By 1969, she became a fellow in the Genetics and Metabolism Department at the University of California. She joined King Faisal Specialist Hospital and Research Centre (KFSHRC) in Riyadh in 1978, where she established one of the first genetic departments in Saudi Arabia (Cadogan, 2020). With a high rate of consanguinity in the population along with prominent genetic disorders among children in Saudi Arabia, the establishment of this Department of Genetics was crucial to unearth the role of genetics in such cases. During her stay in KFSHRC, she held positions as chairman of Pediatrics from 1987 to 1989, and as director of the Genetics/Endocrinology and Metabolic Disease Fellowship Program from 1989 to 1995. Dr. Sakati was the head of the Department of Medical Genetics from 1995 to 2001. Her major breakthrough was the discovery of three rare genetic syndromes that were named after her. Sakati-Nyhan-Tisdale syndrome, a disease she reported in 1971 along with her colleagues William Leo Nyhan and William Tisdale, was described in an 8 year old boy with bone malformations and congenital heart disease (Sakati et al., 1971). Sanjad-Sakati syndrome, another rare genetic condition that was first recorded in Saudi Arabia by Sakati along with Sami A. Sanjad in 1991, is characterized by congenital hypoparathyroidism associated with severe failure to grow along with dysmorphism (Sanjad et al., 1991). This discovery helped with the diagnosis of children showing similar phenotypes and brought a step closer the mapping of rare genetic disorders across Saudi Arabia (Sanjad et al., 1991). Woodhouse-Sakati syndrome, the third rare genetic disorder discovered by Sakati in collaboration with Nichols Woodhouse in 1983, was discovered by investigating seven Saudi patients suffering from hypogonadism, alopecia, diabetes mellitus, mental retardations, and deafness, along with ECG abnormalities (Sakati and Woodhouse, 1983). Her discoveries and work on these rare diseases are summarized in two books she coauthored with William L. Nyhan entitled “Genetic and Malformation Syndrome in Clinical Medicine” (1976) and “Diagnostic Recognition of Genetic Disease” (1987) (Nyhan and Sakati, 1976; Nyhan and Sakati, 1987). Due to Dr. Sakati’s outstanding accomplishments and commitment to genetic research in Saudi Arabia, His Royal Highness, King Fahad bin Abdulaziz granted her the Saudi Nationality in 1993 (Takreem, 2018). In 2001, she was recognized as a distinguished senior consultant in KFSHRC, a position she still holds, and in 2018 she was awarded The Special Distinction Award by Takreem, an organization that aims to honor Arab laureates and recognize their great achievements (Takreem, 2018). During her 40-years tenure at King Faisal Hospital, she trained and educated many Saudi residents and fellows, and her discoveries changed the life of many families in Saudi Arabia by providing hope of having healthy children through chorionic villus sampling and segregation analysis, and by being able to allow families to comprehend the diagnosis of their children with a controlled management and treatment plan.

From Egypt, we want to honor Samia Aly Temtamy, the founder of human genetics in Egypt. Her inspiration to be a physician was seeded when she was only 10 years old. She fell ill with typhoid fever and was treated by Dr. Ibrahim Nagui, whom she subsequently considered a role model and an inspiration to become a doctor. Her passion for medicine grew when she started reading her older brother’s medical textbooks (Temtamy, 2019). She was one of the first female graduates in her class at the Faculty of Medicine at Cairo University in 1957, where then-president of Egypt Gamal Abd Elnasser bestowed her the graduate certificate. She spent 1 year as an intern at Cairo University Hospital, followed by 2 years as a pediatric resident at Cairo University Children Hospital. In 1960, She completed her Diploma in Child Health (D.C.H) from Faculty of Medicine, Cairo University. During her residency, children suffering from various congenital malformations were seen by her-at a time when very limited knowledge and research was available in this area. While accompanying her husband who completed his fellowship training in cancer research at John Hopkins University (Temtamy, 2019), she was accepted into a new PhD program in Human Genetics at Johns Hopkins University. Temtamy earned her PhD degree in 1966 as one of the first Arab females to earn a doctorate in this field (National Research Centre in Egypt, 2021). During her PhD research, she was able to propose for the first time a classification of hand malformations based on anatomical positions of these anomalies and their morphology. She later published a book with her mentor Victor McKusick summarizing her extensive work on hand malformations entitled “The Genetics of Hand Malformation” (Temtamy and McKusick, 1978). In addition to this brilliant nosology, she discovered over 30 syndromes in collaboration with coworkers through patients she investigated (Temtamy, 2019). Several of these syndromes were named after her, including Temtamy syndrome (Temtamy et al., 1996) and Temtamy Preaxial Brachydactyly syndrome (Li et al., 2010). After completing her PhD, she was determined to return to Egypt to establish the field of human genetics in her country and deliver the knowledge she gained during her stay in the US. In 1977, she founded the first human genetics department in the National Research Center. Later, it expanded until it became the Center of Excellence of Human Genetics in 2014. The Center is now comprised of eight departments including Clinical Genetics, Orodental Genetics, Cytogenetics, Prenatal Diagnosis and Fetal Medicine, Biochemical Genetics, Immunogenetics, Medical Molecular Genetics, and Molecular Genetics and Enzymology, with more than 200 researchers enrolled. Dr. Temtamy initiated a national program for neonatal screening and served as its principal investigator. This program screened over 15,000 newborns and identified a high frequency rate of Hypothyroidism and PKU among the Egyptian population (Temtamy, 1998). In 1985, she was appointed the head of the Division of Genetic Engineering and Biotechnology (National Research Centre in Egypt, 2021). In 1995, she became professor Emeritus of Human Genetics at NRC. She was a vital member in the African Society of Human Genetics and the National Society of Human Genetics. In 2017, Dr. Temtamy was invited by the Human Genome Organization (HUGO) and awarded the HUGO African prize for lifetime contributions to human genetics. In Egypt, she was awarded the Nile Prize by the Academy of Scientific Research and Technology in 2011, and the State Prize of Merit in Medical Sciences by the Egyptian Academy of Scientific Research and Technology in 2000 (The women and memory forum, 2018). Unfortunately, in June 2021 Samia Temtamy passed away, leaving behind a treasury of knowledge in genetic diseases. She will be remembered as a role model of an ambitious, successful, and determined woman.

From Jordon hails to Rana Dajani, a scientist and feminist that committed herself to speak up for Arab women and their perspective challenges faced in science. Her impressive scientific journey started at the University of Jordon, where she completed her bachelor’s degree and master’s degree with first honor awards, and then pursued her PhD in molecular cell biology at the University of Iowa in 2005 (HU University, 2021). Being a young mother by that time, her spouse resigned his job to relocate to the States providing the family support during her career progression (Abedalthagafi 2018). Her research focused on cell signaling and inter- and intra-regulatory networks within a cell through interdisciplinary approaches (HU University, 2021). Her research interests also extended to genome-wide associations studies (GWAS) in the fields of diabetes, cancer, and stem cell research. Her research on stem cells initiated the necessity to establish the foundations of stem cell research ethics laws in Jordan (Hauser, 2017). She pleaded for the theory of biological evolution in Islam (Hauser, 2017). She is an international expert on the genetics of Circassia and Chechen populations in Jordan. Currently, Dr. Dajani is a tenured professor of biology and biotechnology at the Hashemite University in Jordan. She has been awarded a 2019–2021 Zuzana Simoniova Cmelikova Visiting Scholar Award at the Jepson School of Leadership Studies at the University of Richmond, the first institution dedicated to leadership education. Prior to this, she has been a fellow at the Radcliffe Institute for Advanced Studies at Harvard University. During her stay at Radcliffe, she dedicated some of her time to publish her book “Five Scarves: Doing the Impossible-If we can reverse cell fate, why can’t we redefine success?” (Dijana, 2018). In her book, she documents her personal journey from growing up and starting a family to being a genetic expert and advocating for biological evolution within an Islam perspective. The title “Five scarves” serves as a reference to her own roles as mother, teacher, scientist, social entrepreneur, and feminist. The book is a brilliant approach to document the hurdles she faced as an Arab Muslim woman in academia, and reveals the different views and women’s experiences in various cultures and religions. Moreover, she wrote several articles in *Nature* about women’s education and struggles in science as part of her enduring efforts for women’s liberation (Dajani, 2012). She also is the president of the Jordan chapter for the Organization for Women in Science for the Developing World, an organization that provides training opportunities for women scientists. She is an advisor for the UN Women Advisory Jordan Council and founded a mentoring program named *The Three Circles* that provides support for Arab females in science (Dajani, 2021a). She received several other honors as well, including the Eisenhower and two Fulbright fellowships, and was invited as visiting professor to Yale University and as a visiting scholar to Cambridge University (Dajani, 2021b). In 2015, She was chosen by *Arabian Business* to be among the 100 most powerful Arab women in science and healthcare (Arabian Business, 2015). Beyond being a trailblazer for women, Dajani established a non-governmental organization known as *We Love Reading*–an idea born upon her return to Jordan from the United States where she realized the scarcity of community libraries in Jordan. She started by recounting weekly story sessions for children in her community mosque that later evolved to establish an organization to nourish the love of reading in children from a very young age. Her essential dogma is that reading is a powerful tool that every child needs to conquer under any circumstance. This program has trained around 700 women to be storytellers, built more than 300 community libraries across Jordan and refugee camps, and distributed more than 250,000 books worldwide reaching as far as Mexico, Turkey, Thailand, Azerbaijan, and Uganda (Arabian Business, 2015). Dr. Dajani earned numerous awards, including the UNHCR Nansen Refugee Award for the Middle East, the Synergos Award for Arab World Social Innovators and a WISE award. Nationally, she was honored with the Order of Al Hussein for Distinguished Contributions of the Second Class in 2014, earned by those who made distinguished contributions to the Jordan society. She was acknowledged by his Majesty King Abdullah II of Jordan as a women leader in 2015 (Dajani, 2021a).

## Summary

Throughout history, powerful women changed the course of events, reformed policy decisions, asserted their stance and defied the status quo. Queens Cleopatra of Egypt and Victoria of the United Kingdom played central historic roles (Froelich, 2020). At the same time, however, women that have not been fortunate enough to have a royal or upper-class heritage have very often been subjected to gender inequality, cultural and societal hurdles, and experienced countless difficulties imposed on their education and careers (Andres, 2011). This review, aimed to specifically recognize and celebrate non-western women in the field of genetics, attempts to shed light on the life stories and outstanding accomplishments of female scientist that did not accept the stereotypical roles that society had tried to force on them. The selected examples presented here show what non-western women scientists have been able to accomplish. Our selected heroines with diverse backgrounds, each was vulnerable to at least a form of barrier. Narry Kim sets an example of a rigorous woman that currently holds a managerial appointment confronting the fact of women’s under-representation in high ranked positions in East Asia (World Economic Forum, 2020). Meanwhile, Thailand is one of the South-East countries that approached the finish line to gender equity in several fields like heath sector (Bekhouche, 2013), the limited fund availability for biotechnology research imposes a hurdle to young female researchers (Nogrady, 2019). Chanchao Lorthongpanich, devoted her research to find cost effective alternatives to resolve blood donation issue in Thailand since thriving in an equally compelling environment never fails to foster women that could make an enormous difference. The impact of adopting potent laws to protect women’s rights, governmental imperatives to support women’s inclusion in labor workforce is implemented in Singapore. We could relate the rising star Yue Wan, as an example. Prior approaching her mid-thirties, she led a team as a junior PI pioneering in innovative sequencing technology to decipher RNA functional role. On Contrary, we witnessed Adeyinka Falusi’s tenacious efforts to pursue her career in a lower women’s supportive environment. Her primitive support was based solely on family and spouse. We could categorize older generation examples, Archana Sharma, Nadia Sakati, and Samia Temtamy in a category of conquering the ideology of male dominating society by introducing a new field of science in their respective country. A pressing stance for women’s contribution to science when provided the right resources for knowledge and education. Archana had a vital role to India’s national development post the independence and setting a blueprint for botany education globally (Khan 2020). Nadia’s knowledge and staunch research permitted the discovery of the Saudi genetic profile distinctiveness and establishment of medical genetics in Saudi Arabia (Takreem, 2018) while Samia is the main contributor of founding the center of excellence in human genetics in Egypt (Temtamy, 2019).Our last selected scientist, Rana Dajani, sets a triumphant image of modern muslim women in science. Besides, advocating to abolish the hijab stereotype as an indication of oppression in western communities (Dajani, 2012). She conveyed modesty for evolution and stem cell research in Islam. An eccentric challenge that would only be rectified by empowered education. Resembling the husband’s support Adeyina Falusi acknowledged, Dajani’s spouse support during her graduate studies was a key for her career nourishment. Being descendant of Palestinian and Syrian parents, she explicitly promoted for educational and mentorship programs in Arab world and in particular refugees’ campuses. We believe our selection, however limited, could provide an improved understanding of the diverse hurdles experienced in non-western countries for women. Confronting the society determinants, struggling a family work balance, acceptance of evolution in a religious setting, introducing an ideology of a female favorable environment and many more. We can point out three influential factors that is common among most of our selected scientists: 1) the fundamental impact of travelling abroad for education. 2) The motivated resilience experienced upon family/spouse support. 3) Achieving a balanced work-family or female positive setting. We summarized our listed scientists and the influential factors affecting their career progression weather it is a challenge or a support, a limited attempt to comprehend women’s position in these regions (Table 2).

**TABLE 2:** Summarizing our selected scientists and some of the influential factors that affected their career progress.

Female scientists	Region	Selected factors
Narry Kim	East Asia	Under representation of women in managerial and high ranked positions in East Asia
Chanchao Lorthongpanich	Southeast Asia	Gender balanced environment paving the road to address and find solutions for national issues
Yue Wan	Southeast Asia	Gender balanced environment in workforce/laws protecting women’s right
Archana Sharma	South Asia	Thriving in a male dominated society
Adeyinka Falusi	West Africa	Under representation of women in research/Poverty/Religious/Cultural factor/Family support
Nadia Sakati	Middle East	Thriving in a male dominated society/Promoting employment opportunity
Samia Temtamy	Middle East	Thriving in a male dominated society/Challenging employment opportunity
Rana Dajani	Middle East	Muslim women Stereotyping/Modesty in Science/Family support

Today the world’s advancement is driven by progress in science and technology. Many of our most pressing problems, including global warming, viral pandemics, strained energy resources and environmental pollution can effectively only be solved by advances in science and technology (UNESCO Report Science, 2021). For this, we must combine and harness intellectual power of all humans, and hence it is imperative that women must be part of this process. Specifically, the female population in the non-western world represents a vast reservoir of intellectual potential that has not been allowed to effectively contribute to scientific and technological advances. To change this dilemma, women must be empowered and enabled to have the same education paths and career choices available to them as their male counterparts. National and international initiatives need to promote women for managerial and leadership positions, and not stop at ensuring only basic education for young girls. This change will necessitate an ongoing reformation of social and societal attitudes to enable women to thrive in education and career. While much more needs to be done, governmental entities and science institutes in non-western countries has been taking important steps to enable women’s access to equal rights, education, and career opportunities.

With the immense knowledge gained by the sequencing of the human genome, the deciphering of cellular signaling pathways in normal and disease states, and the development of novel therapeutic approaches, genetics and related fields are at the forefront of a medical revolution of an unprecedented magnitude. Ethical and legal concerns associated with this progress will need to be addressed on an ongoing basis. Steering and controlling this scientific and medical revolution and associated regulatory issues will be more difficult without considering women a pillar of our science community and the establishment of a supportive environment encouraging more women to be involve in STEM.
